# Machine learning prediction‐informed gaze optimization in ocular proton therapy with NTCP evaluation

**DOI:** 10.1002/mp.70108

**Published:** 2025-11-08

**Authors:** Daniel Björkman, Antony Lomax, Maria De Prado, Alessia Pica, Jan Hrbacek

**Affiliations:** ^1^ Center for Proton Therapy (CPT) Paul Scherrer Institute Villigen Switzerland; ^2^ Department of Physics Eidgenössische Technische Hochschule (ETH) Zürich Switzerland

**Keywords:** automation, intra‐ocular tumors, radiotherapy, treatment planning

## Abstract

**Background:**

Ocular proton therapy (OPT) treatment planning has remained largely static since the introduction of the EyePlan system over four decades ago, which is still widely used across treatment centers. The current method relies on planners individually selecting gaze fixation points to optimize eye orientation, aiming to minimize the exposure to sensitive sensitive ocular organs at risk (OARs). However, this manual process lacks explicit information on how eye orientation correlates with toxicity risks. This limitation presents an opportunity to enhance planning efficiency, reduce planner biases, and provide toxicity‐informed guidance through automated computational tools.

**Purpose:**

The primary objective of this research is to develop a computationally efficient automated approach for OPT treatment planning that optimizes eye orientation during treatment. By providing intuitive and clinically relevant information to treatment planners, the approach aims to minimize healthy tissue exposure and reduce the risk of radiation‐induced toxicities.

**Method:**

Dose calculations are conducted using the in‐house developed treatment planning system (OCULARIS) guided by machine learning (ML) gaze predictions and a tailored cost‐function that allows to quickly identify eye orientations with favorable healthy tissue sparing suitable for treatment. The orientations identified by the optimization algorithm are subsequently evaluated against the clinically defined orientations, utilizing both cost metrics and recent normal tissue complication probability (NTCP) models, acknowledging the inherent uncertainties associated with these models. The resulting plans are then validated and evaluated by two clinically experienced medical physicists.

**Results:**

In 92% of patient cases, the automated plans were found to be preferable or comparable to the clinical plans, with 25% rated as preferable. In 67% of cases, the automated plan demonstrated comparable toxicity levels to the clinical plan. Additionally, 47% of cases showed that the automated plans achieve at least a 1% improvement in one of the investigated NTCPs without significantly impacting the others.

**Conclusions:**

This work signifies a more informed strategy for OPT treatment planning, enhancing both speed and automation while reducing planner biases. The methodology offers treatment planners with direct and intuitive information correlating the orientation of the treated eye with toxicity risks. Our investigation considering the direct application of NTCP models demonstrates that eye orientations for OPT could be automatically generated with a comparable or superior treatment suitability for the 92% of investigated patients.

## 1 INTRODUCTION

Proton therapy for intra‐ocular tumors presents several advantages over brachytherapy and LINAC‐based alternatives. The advantage comes mainly from improved 5‐year tumor control and eye retention.^1^, ^2^, ^3^, ^4^, ^5^, ^6^ Treatment planning in ocular proton therapy (OPT) has remained largely unchanged since the introduction of the EyePlan^7^ treatment planning system (TPS) over four decades ago. EyePlan has been the TPS of choice in most dedicated ocular treatment centers worldwide for over 40 years, and in 2016, when an international survey was performed, have been utilized for over 90% of OPT patients to date.^2^ In this context, treatment planners select a gaze fixation point to establish an appropriate eye orientation for isocentric treatments, with the objective of ensuring target coverage while minimizing dose to delicate eye structures. The current workflow of investigating gaze fixation points individually, and the absence of explicit information regarding the correlation between the orientation of the eye and its associated risks of toxicities to the patient, signifies opportunities for improvement. Providing planners with improved tools and additional relevant information through automated computational procedures has the potential to enhance treatment plan quality, reduce clinical biases and promote a more informed treatment planning strategy to mitigate toxicity risks. Notably, a previous exhaustive search approach to automated gaze fixation point identification, as employed by Hennings et al.,^8^ relied on discrete thresholds without accounting for the correlation between eye orientation and toxicity risk. Moreover, EyePlan feature a simple gaze optimization feature which is limited to single objectives and lack important balancing between OARs and as well require manual azimuth selection.^7^ Furthermore, EyePlan offers a simplified manual gaze optimization feature. However, it is limited to single objectives, lacks importance balancing between OARs, and requires manual azimuth selection. The duration of the planning process scales with the quantity of gaze fixation points investigated by the planner, with the duration most commonly taking 15–30 min.

Within the passive scattering OPT therapeutic framework, patients commonly receive a prescribed dose of 60 GyRBE delivered over four fractions with each fraction, lasting 45 to 60 s in the OPTIS2 treatment room at the Paul Scherrer Institute (PSI). For protons, GyRBE is calculated by multiplying the physical dose (Gray) by 1.1. The target volume is defined to follow the eye's surface curvature, incorporating a specific height to approximate the volume outlined by the referring ophthalmologist. Guided by the ALARA (As Low As Reasonably Achievable) principle, the dosimetric field is optimized to minimize radiation‐induced toxicities to surrounding healthy tissues. Note that target coverage is a hard constraint in OPT planning. It is ensured by the selected extent of the SOBP and a tumor‐specific aperture, provided that the projected tumor shape, extended by a lateral margin of 2.5 mm, fits within the 35 mm diameter aperture limit and proton range plus distal margin does not exceed the maximum range of 70 MeV protons (35 mm in water). As such, OAR sparing can only be achieved through selection of the gaze angle. The major treatment objective is therefore the reduction of dose to healthy tissues, with particular emphasis on reducing dose to the macula, optic disc and anterior eye segments. The focus on the optic disc and macula stems from their increased associated risk of optic neuropathy or neovascular glaucoma (NVG),^9^ and the potential need for enucleation from radiation induced complications.^10^ Additionally, it is important to reduce radiation dose to the anterior segments of the eye to minimize associated risk of developing NVG.^11^ This priority is reflected in both our clinical practice and the multidisciplinary consensus at the Erasmus Medical Center Cancer Institute, Netherlands.^12^ Recent analyses correlating dose‐volume metrics for various ocular structures with induced toxicity risks^13^, ^14^ highlight the specific dose‐volume points that require optimization to minimize secondary toxicity in patients. Consequently, this research combined, with our in‐house OPT TPS, OCULARIS,^15^, ^16^, ^17^ facilitates the numerical identification of a suitable eye orientation for treatment.

The objective of this work is to autonomously identify eye orientations that approximate the optimal orientation based on therapeutic objectives, with the intent of enhancing the efficiency of the planning process and rendering it less dependent on the expertise of the planner. Additionally, the objective is to provide planners with supplemental normal tissue complication probability (NTCP) data and a clinically relevant cost‐function to inform and guide clinical decision making. These goals were achieved by incorporating dose points associated with an increased risk of radiation‐induced toxicity, as identified in refs. [13, 14]. The NTCP values were computed from their correlation with these critical dose points, and the same points were employed as variables within the penalty cost‐function. All dose calculations were performed using our in‐house dose engine. It is important to recognize that the NTCP models employed in this study possess inherent uncertainties but represent the best available estimates at the time of analysis. The flexibility of the methods developed in this work enables the incorporation of updated parameters as new data become available. In addition, machine learning (ML) has been utilized to minimize the required computational time by initially predicting a gaze fixation point as a starting point for the optimization procedure. Dose distributions are then subsequently analyzed for the probability of radiation‐induced side effects. A preliminary version of this work was presented at the XXth International Conference on the Use of Computers in Radiation Therapy (ICCR).^18^

## 2 MATERIALS AND METHODS

This section summarizes the automated treatment planning procedure, detailing each methodological component and the associated evaluation strategy. The procedure employs a guided exploration of possible gaze directions, starting from an initial ML prediction. The space close to the prediction is sampled more densely than remote areas, focusing on the most promising space while reducing computational cost. The cost function is subsequently evaluated across all sampled candidates, and the gaze angle corresponding to the lowest cost is identified as the most suitable for treatment. Continuous 2D maps are constructed by interpolating the discrete evaluations, providing smooth representations of either the cost value or the NTCP associated with each monitored toxicity endpoint. Both the cost function and NTCP models employed in this study are based on recent advances in dose‐response relationships for secondary radiation‐induced toxicities following OPT.^13^, ^14^

### 2.1 Machine learning algorithm

Calculating the dose for each eye orientation can be a computationally expensive endeavor. Therefore, an ML algorithm is employed to reduce the necessary search area in which to more densely sample the space of attainable gaze fixation points. The function predicts gaze fixation points from extracted geometric features of the patient's anatomy as represented by the patient model. The gaze fixation prediction function is comprised of a set of random forest regression algorithms, implemented using the open‐source scikit‐learn library,^19^ trained on data representative of the anatomical and treatment variability observed in patients treated at our facility. Random forest regression, an ensemble learning method that combines the outputs of multiple decision trees to improve predictive accuracy and reduce variance, was chosen for its ability to capture complex non‐linear relationships while maintaining robustness to noise and overfitting. The algorithms were trained to approximate the gaze fixation points specified in clinical treatment plans. The final prediction is calculated as the mean output of the algorithm set, enhancing the model's robustness against uncertainties arising from the stochastic variations among the sub‐algorithms consisting of 50 individual decision trees.

The features, represented by their spatial coordinates or scalar distances, include characteristics such as target boundaries, the center of mass of the target, and the proximity of the target to critical eye OARs. Preliminary investigations assessed the potential contribution of additional patient‐specific variables—including eye laterality (right or left), clinical diagnosis, and patient age—to enhance predictive performance. These factors demonstrated negligible impact on model accuracy and were therefore excluded from the final feature set.

#### 2.1.1 Training dataset

The training dataset, comprised 1730 patients, representing all patients treated with OPT at our institute between 2011 and 2023. This comprehensive cohort captures a wide range of anatomical variability, tumor locations, and gaze directions encountered in clinical practice, ensuring that the predictive model was trained on a representative and diverse set of treatment scenarios.

#### 2.1.2 Cross‐validation protocol for model evaluation

Model performance was assessed using a leave‐one‐out cross‐validation strategy, whereby each patient's gaze prediction was computed using a model trained on all remaining patients of the training dataset. The held‐out patient was explicitly excluded from the training data to provide maximal training data per prediction.

### 2.2 Eye modeling and dose calculation

**FIGURE 1:**
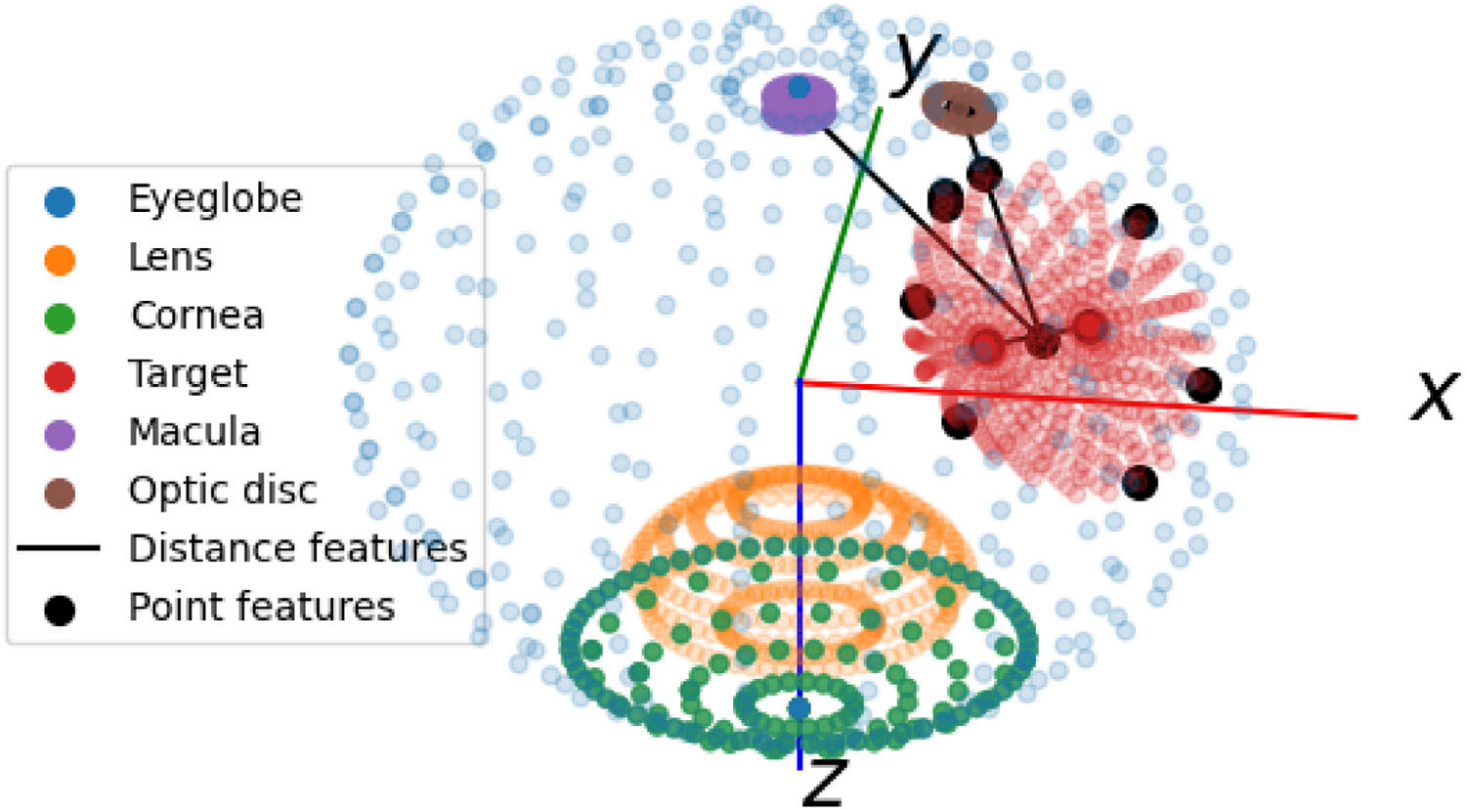
Point cloud patient model exported from EyePlan in the gaze centered reference frame with highlighted features for ML gaze fixation point prediction. ML, machine learning.

The EyePlan derived 3D point cloud model (Figure 1) includes the target and organs at risk (OARs) such as the lens, retina, ciliary body, cornea, macula, optic disc, as well as the entire eye globe. Additionally, a subset of geometric features used in the ML‐based gaze prediction model is visualized. The orientation of the eye model is determined by its rotation point and the direction of its gaze vector, in accordance with the framework described in ref. [15].

For this work, dose calculations from OCULARIS have been integrated with the 3D point models of the patient exported from EyePlan treatment plans to represent dose depositions of the passive scattering proton delivery in OPTIS2. These point clouds served a dual purpose: they enabled surface identification via ray‐tracing techniques and supported dose attribution by mapping calculated dose values to voxels corresponding to binary mask representations of individual anatomical structures. All dose calculations are examined on a grid comprising 60 × 60 × 60 voxels, each with a resolution of approximately 0.59 mm/voxel, similar to the grid parameters in the corresponding clinical treatment plan defined in EyePlan. OCULARIS calculates 3D dose distributions as a function of proton range, modulation length, patient surface information and target shape. Both proton range and modulation length are derived from target and patient surface models, while the projection of the target shape corresponds to the lateral inflection of the fall‐off, characterized as a sigmoid function. The dedicated dose engine module of OCULARIS adapts the principles outlined in Equation (5) of ref. [20], integrated into an object‐oriented framework with design decisions drawing inspiration from the matRad system.^21^ Specifically, the program was designed similarly to prioritize ease of customization and accessibility through a scripting interface. OCULARIS has undergone thorough validation through comparative analysis with EyePlan and the comparison to measured patient‐specific depth dose profiles across a cohort of 50 patients.^17^, ^22^ The OCULARIS source code has been made accessible to the OPT community and can be found at.^16^

### 2.3 Gaze angle sampling

**FIGURE 2:**
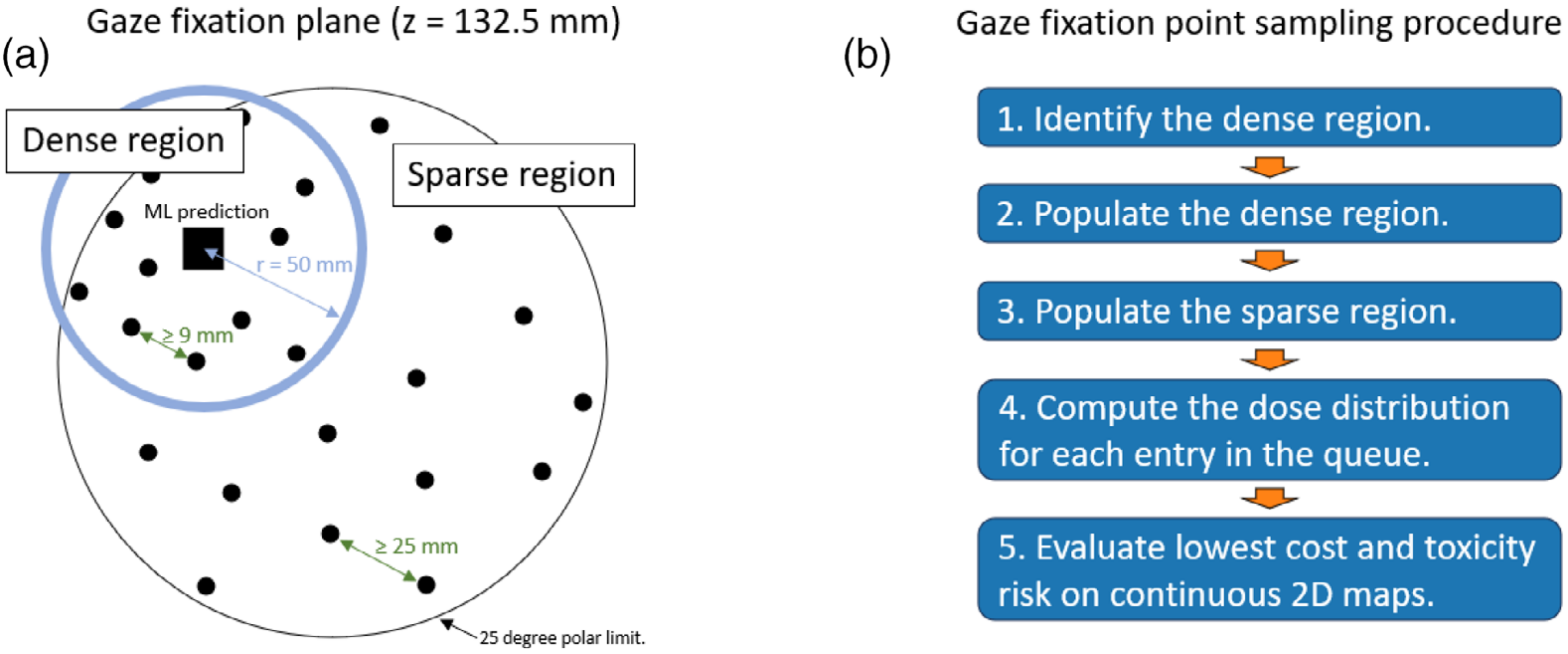
(a) Delineation of the dense and sparse gaze fixation point sampling regions as defined by the ML prediction. (b) The gaze fixation point identification procedure involves a directed sampling centered on the machine learning prediction, followed by gaze fixation space sampling and subsequent cost and NTCP evaluation. ML, machine learning; NTCP, normal tissue complication probability.

The gaze sampling procedure is designed to efficiently explore the gaze fixation space, with the density and ordering of samples biased toward regions most likely to contain the optimal fixation point. The region of increased sampling density is defined by the ML prediction, while a first in, first out queue data structure determines the sequence in which candidate orientations are evaluated. Each orientation corresponds to a gaze fixation point for which the target is aligned to the isocentre. These orientations are systematically sampled across a defined angular space: polar angles from 0 to 25 degrees, and azimuthal angles from 0 to 360 degrees, ensuring comprehensive coverage of clinically feasible gaze directions. Fixation points are randomly drawn from within this space, using spacing constraints to avoid redundant sampling. In this study, the denser and less dense sampling regions are separated by 9 and 25 mm, respectively, within the gaze fixation space located 132.5 mm upstream of the isocentre, as illustrated in Figure 2a. The sampling density was chosen to ensure meaningful separation between points in the vicinity of the gaze direction indicated by the ML prediction, while the remaining space is sparsely sampled to achieve satisfactory coverage of the gaze fixation space. This approach strikes a balance between thorough exploration of the space and computational efficiency.

Building on this rationale, the procedure is implemented in five sequential steps, as illustrated in Figure 2b. Step 1 is to identify the denser region using the ML model, which provides the initial localization of the region in which the most suitable fixation point is likely located. In this study, the denser region is defined as extending 50 mm from the ML prediction. Step 2 is to populate the candidate queue of gaze fixation points with entries drawn from this denser region, prioritizing those most likely to be suitable for treatment. To guide the optimization efficiently, the queue is seeded with a denser sampling of gaze directions clustered around the ML‐predicted fixation point, typically situated near the boundary of the clinical polar angle limit. These initial entries represent the most likely treatment‐viable fixation points and are therefore investigated first. Step 3 is to populate the candidate queue with fixation points sampled from the remaining sparser region, continuing until the region is fully covered. Although less critical, these points are included at a lower sampling density to expand coverage of the fixation space and to account for uncertainty in the ML predictions. Step 4 is to systematically compute the 3D dose distribution for each candidate entry in the queue, proceeding sequentially until all entries have been processed or the simulation is terminated. Finally, Step 5 is to evaluate all the resulting orientation candidates on continuous two‐dimensional maps of the gaze fixation plane. Each orientation is defined by its transversal *x*‐ and *y*‐coordinates together with its polar and azimuthal angles, and is color‐coded according to the cost function or NTCP model under investigation. This enables identification of the orientation that minimizes cost while reducing toxicity risk.

### 2.4 Gaze optimization

In the realm of OPT, treatment planning involves identifying the gaze fixation point corresponding to the eye orientation with optimal sparing of healthy eye OARs for the patient. The objective of the optimization can therefore be formulated with Equation (1) as:

(1) $$\underset{O}{\text{minimize}}\;C\left(O\right)$$

Where:

$$\begin{array}{cc} & O\;\text{represents an attainable eye orientation for treatment,}\\ & C(O)\;\text{is the cost function representing the quality of the}\\ & \;\text{treatment plan for orientation}\;O,\;\text{and is calculated}\;\\ & \text{from the dose distribution estimated by our dose engine.} \end{array}$$

#### 2.4.1 Cost function for healthy tissue sparing

The cost‐function, as defined in Equation (2), attempts to emulate the decision‐making process of the planner. It has been formulated to prioritize the minimization of specific dose points that are associated with a heightened risk of inducing secondary toxicities in vulnerable eye OARs. Target coverage is not incorporated into the cost function, as it is guaranteed by the choice of SOBP extent for the treatment field and the patient specific aperture. The cost‐function calculates C by applying weighted penalties to the dose attributed to healthy OARs of the eye as follows:

(2) $$\begin{array}{cc} C= & \sum_{i=1}^{N}\frac{w_{i}}{M}\sum_{j=1}^{M}Dose_{ij}+\\ & w_{macula}\cdot D_{2{\%}_{macula}}+\\ & w_{opticdisc}\cdot D_{20{\%}_{opticdisc}}+\\ & w_{cornea}\cdot D_{20{\%}_{cornea}}+\\ & w_{retina}\cdot V_{55GyRBE_{retina}}+\\ & w_{ciliarybody}\cdot V_{27GyRBE_{ciliarybody}}+\\ & w_{lens}\cdot D_{5{\%}_{lens}} \end{array}$$

Where:

$$\begin{array}{cc} & Dose_{ij}\;\text{represents the dose-volume points for the}\;\\ & i\text{-th OAR and}\;j\text{-th volume point,}\\ & N\;\text{is the total number of considered eye OARs,}\\ & M\;\text{is the number of dose-volume points considered for}\\ & \text{each OAR (e.g., V5, V10, …, V100),}\\ & w_{i}\;\text{is the tunable weight assigned to the}\;i\text{-th OAR.} \end{array}$$

In formulating this cost‐function, a deliberate strategy has been employed to preserve healthy tissue by assessing dose to the most clinically relevant ocular OARs of the patient model. This strategy is informed by recent findings on dose‐response to healthy tissue identified by Espensen et al. and Thariat et al.^13^, ^14^ The dose‐volume points associated with the retina and ciliary body were adjusted by a factor of 0.96 to account for the slight difference in fractionation scheme. The corresponding NTCP curves were scaled accordingly. This factor adjusts the results of Espensen et al. to account for differences in fractionation schemes, aligning them with the PSI protocol to ensure an equivalent biological effective dose (BED). The relative biological effective dose, $BED_{4\times 14.3\;\text{GyRBE}}/BED_{4\times 15\;\text{GyRBE}}$, is calculated using the formula $BED=nd\left(1+\frac{d}{\alpha /\beta }\right)$, where n represents the number of fractions, d the dose per fraction, and α/β=1 representative for ocular tissue.^23^ Consequently, the values presented in this work are therefore expressed for the 4 × 15 GyRBE fractionation scheme practiced at PSI.

The first term of the cost‐function imposes weighted penalties on the area beneath the dose‐volume histogram curves for the selected ocular OARs, aligning with the clinical objective of minimizing radiation exposure to healthy tissue. The second to sixth terms penalize $D_{2{\%}_{\text{macula}}}$, $D_{20{\%}_{\text{optic disc}}}$, $D_{20{\%}_{\text{cornea}}}$, $V_{55GyRBE_{retina}}$ and $V_{27GyRBE_{ciliarybody}}$ respectively. These dose‐volume points were selected due to their importance in being predictive for maculopathy, optic neuropathy, NVG, secondary ischemic retinal detachment and cataract respectively as outlined in Espensen et al.^13^ Notably, when the volume of the retina receives close to 100% of the prescribed dose the risk of retinal detachment following treatment is substantially increased.^13^ The seventh term is formulated to impose a penalty on the $D_{5{\%}_{lens}}$ as it is associated with increased risk of vision‐impairing cataracts (VIC).^14^

In this study the macula and optic disc were considered the most critical OARs and were therefore assigned a weighting factor of 3 whereas all other OARs were assigned a weighting factor of 1.^12^ The specific values were established during the prototype phase, demonstrating promising outcomes on a limited patient cohort prior to applying the weighting parameters to this broader study. Further optimization of the weighting parameters could be explored and in a clinical setting they would likely be determined through a user interface guided by accumulated experience over time. Consequently, the weights are not tailored to any specific patient. Precise optimization of the weights is considered beyond the scope of this study, as any modifications to the cost function would require a comprehensive re‐evaluation of the specific weights. The determination of these weights is best guided by clinicians, leveraging their expertise and clinical experience, rather than being rigidly defined at this stage of research. To align with this perspective, a straightforward approximation of the weights was employed, designed to reflect the clinical workflow while maintaining focus on the broader objectives of the study.

### 2.5 NTCP modeling

The relationship between ocular orientation during treatment and the incidence of secondary radiation‐induced toxicities was quantified by evaluating dose metrics in the corresponding treatment gaze direction, as listed in Table 1. These dose points were then correlated with the probability of specific NTCP, as identified by Espensen et al.^13^ While these NTCP estimates were not explicitly included in the cost‐function, they served as a supplementary metric during evaluation to provide planners with interpretable toxicity risks.

**TABLE 1:** Dose‐volume metrics and associated secondary radiation‐induced toxicities.^13^

OAR	Endpoint	NTCP metric
Macula	Maculopathy	$D_{2\%}$
Optic disc	Optic neuropathy	$D_{20\%}$
Cornea	Neovascular glaucoma	$D_{20\%}$
Retina	Retinal detachment	$V_{55GyRBE}$
Ciliary body	Cataract formation	$V_{27GyRBE}$

### 2.6 Patient data

The complete framework of this study is evaluated on a cohort of 36 patients who underwent OPT at the Centre for Proton Therapy, PSI, Switzerland, between 2010 and 2023. The corresponding treatment plans were defined using EyePlan, with each plan incorporating the positions of fiducial tantalum clips, which are used to register the patient‐specific eye model to the treatment position. The plans were retained exactly as originally defined for clinical application. The patient dataset was selected to encompass proton ranges spanning from 11 to 30.7 mm, the full range of treated gaze angles and varied anatomical geometries to reflect the diverse scenarios treated in OPTIS2. A total of 23 patient cases involved targets located posterior to the eye equator (PE), while 13 cases had targets extending across both the anterior and posterior regions of the eye equator (AE/PE), including 3 cases where the targets extended through the ciliary body. One patient was diagnosed with hemangioma (PE), while the remaining cases were treated for uveal melanoma. Specifically, the dataset encompasses a spectrum of ocular metrics tabulated in Table 2. Patients who underwent treatment with a wedge compensator were excluded from the study, as the wedge's position requires optimization subsequent to determining the treatment orientation.

**TABLE 2:** Anatomical variation in patient cohort.

Feature	Range	Unit
Target height	2.35–11.50	mm
Target base area	34.20–671.65	${\mathrm{mm}}^{2}$
Target volume	57.67–2795.21	${\mathrm{mm}}^{3}$
Distance from target to optic disc	0.08–16.21	mm
Distance from target to macula	0.41–15.69	mm
Distance from target to ciliary body	0.02–18.67	mm

### 2.7 Treatment plan comparison

The cohort‐wide analysis comparing automated and clinical treatment plans is divided into two distinct components: a non‐blinded preference review and a numerical NTCP analysis. The non‐blinded preference review was carried out by two medical physicists, who evaluated and reached a consensus while being aware of which plan was the clinical plan. Each physicist recorded their preference regarding the alignment of the plans with the treatment objectives. This assessment was based on clinically relevant dose–volume metrics, with physicists directly evaluating two‐dimensional maps displaying cost or NTCP values as a function of gaze angle. In general, preference was given to plans that demonstrate a potential reduction in NTCP for any OAR, provided that the remaining toxicities did not worsen. In cases where trade‐offs between multiple toxicities occurred, or where specific clinical constraints took precedence over NTCP reduction, the physicists incorporated these factors into their final judgment. The numerical NTCP analysis includes the classification of patient cases into four distinct categories based on calculated toxicities: improved, neutral, trade‐off, and inferior. This classification process was facilitated by using a 1% threshold to filter out low magnitude NTCP differences. That is, a plan was considered improved if at least one toxicity showed an absolute reduction of at least 1%, while ensuring that no other toxicities experienced an absolute increase of 1% or more. A plan was categorized as neutral if the difference between the clinical and automatic plan were within 1%, indicating that the plans exhibit a comparable degree of alignment with the treatment goals. In contrast, a plan is identified as inferior if at least one toxicity worsens by at least 1%, while the other toxicities remain within the same threshold. Finally, a trade‐off plan is characterized by a scenario where some toxicities improve while others deteriorate by at least 1%.

## 3 RESULTS

### 3.1 Gaze angle comparison

**FIGURE 3:**
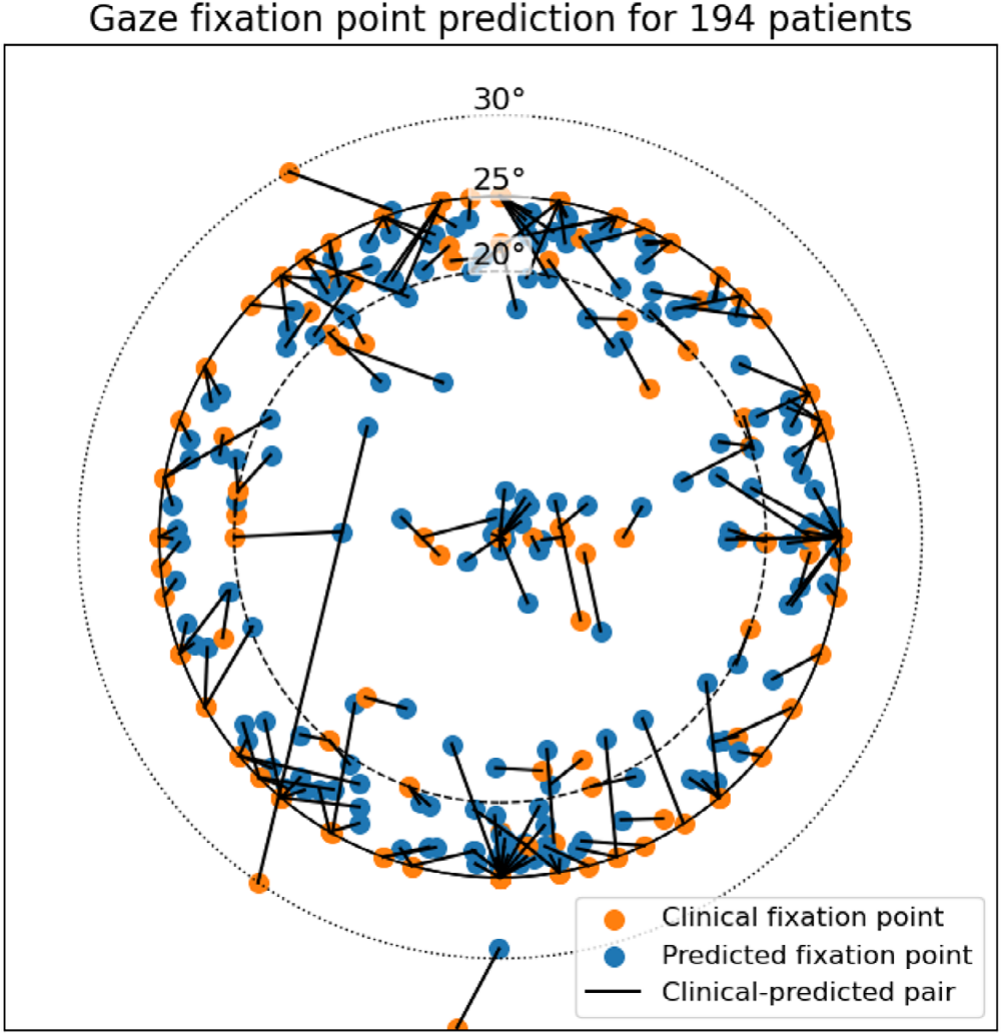
Machine learning predictions of gaze fixation points, represented in polar and azimuthal coordinates, are mapped on a plane located 132.5 mm anterior to the isocenter from the patient's perspective. This analysis includes data from 194 patients, with corresponding clinical fixation points connected to the ML predictions by black lines to illustrate their correspondence.ML, Machine learning.

The ML predictions closely correspond to the clinical orientations, as illustrated in Figure 3, based on a representative subset of 194 patients—approximately 10% of the full training dataset. The predictions exhibit a small underestimation of the polar angle, deviating by no more than 5 degrees in 75% of the predictions on the full training set. The black lines in the figure illustrates the proximity between the predicted fixation point and the clinical fixation point. The features exerting the most significant influence on the prediction of the gaze fixation point is the center of mass of the target in relation to the center and rotation point of the model in the gaze centered reference frame.

The cost minima were located within the densely sampled region identified by the ML prediction in 29 (92%) out of 36 patient cases. In the remaining 7 (19%) cases, the minimum‐cost solution was found outside the predicted region. One case (3%) showed a clear discrepancy between the ML prediction and the cost minimum. In five cases (14%), the clinically applied solution fell outside the ML‐predicted region.

**FIGURE 4:**
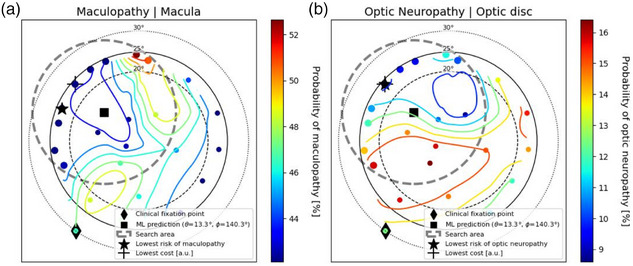
Investigated fixation points for an example patient color‐coded based on their respective probability of maculopathy (a) or probability of optic neuropathy (b).^13^ The gaze fixation point with lowest cost is associated with the lowest risk of optic neuropathy for this patient. The target of this patient is situated in the right eye, located nasally, inferiorly, and posteriorly relative to the center of the eye. Larger markers denote key candidate gaze fixation points for treatment.

**FIGURE 5:**
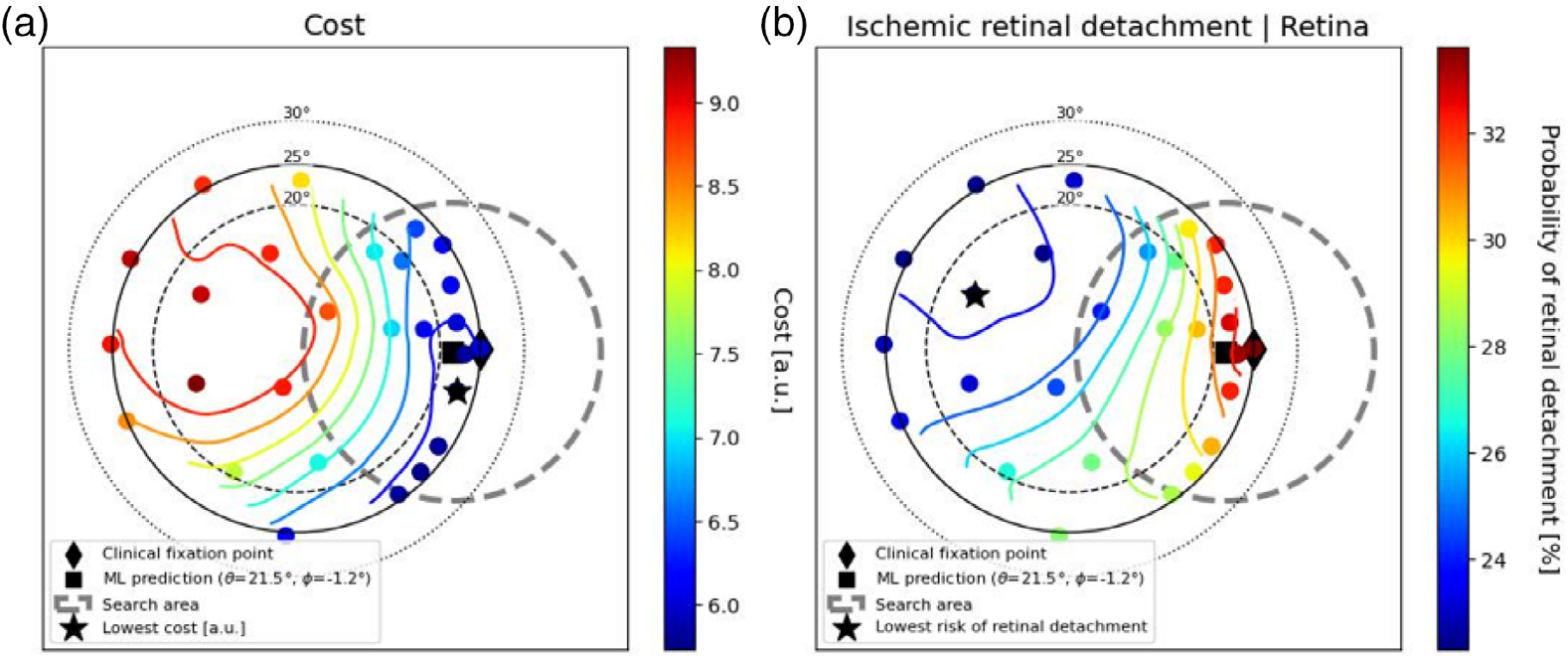
Investigated fixation points for an example patient color‐coded based on their respective cost (a) and probability of secondary ischemic retinal detachment (b).^13^ The target of this patient is located in the right eye near the posterior pole, positioned temporally and superiorly relative to the center of the eye. Larger markers denote key candidate gaze fixation points for treatment.

Figures 4 and 5 present examples from two patients, with an additional two cases provided in Section A.1. These cases were selected to either illustrate scenarios with potential for treatment plan improvement or to demonstrate the alignment between the cost function and NTCP outcomes. In each subplot, the explored fixation points are color coded depending on their cost or NTCP values.^13^ Both figures show gradients which slope towards favorable healthy tissue sparing, suggesting strategies to modify the gaze angle for reducing radiation exposure to health tissue. Figure 4 highlights the patient case with the greatest potential for improvement, demonstrating a reduction of at least 2% in the probability of each investigated toxicity. The auto solution is associated with a 4% reduction in the risk of both maculopathy and optic neuropathy, as well as a 5% reduction in the probability of cataract formation (not shown in the figure). The gaze fixation point determined by the automated solution demonstrates a superior alignment with the treatment objectives for this patient. Figure 5 illustrates a patient case demonstrating an optimization strategy to reduce the risk of secondary ischemic retinal detachment by 1.4% through a 15‐degree reduction in the azimuth angle. This adjustment shows similar sparing to other ocular OARs, as indicated by a similar reduced cost penalty.

### 3.2 Treatment plan comparison

**FIGURE 6:**
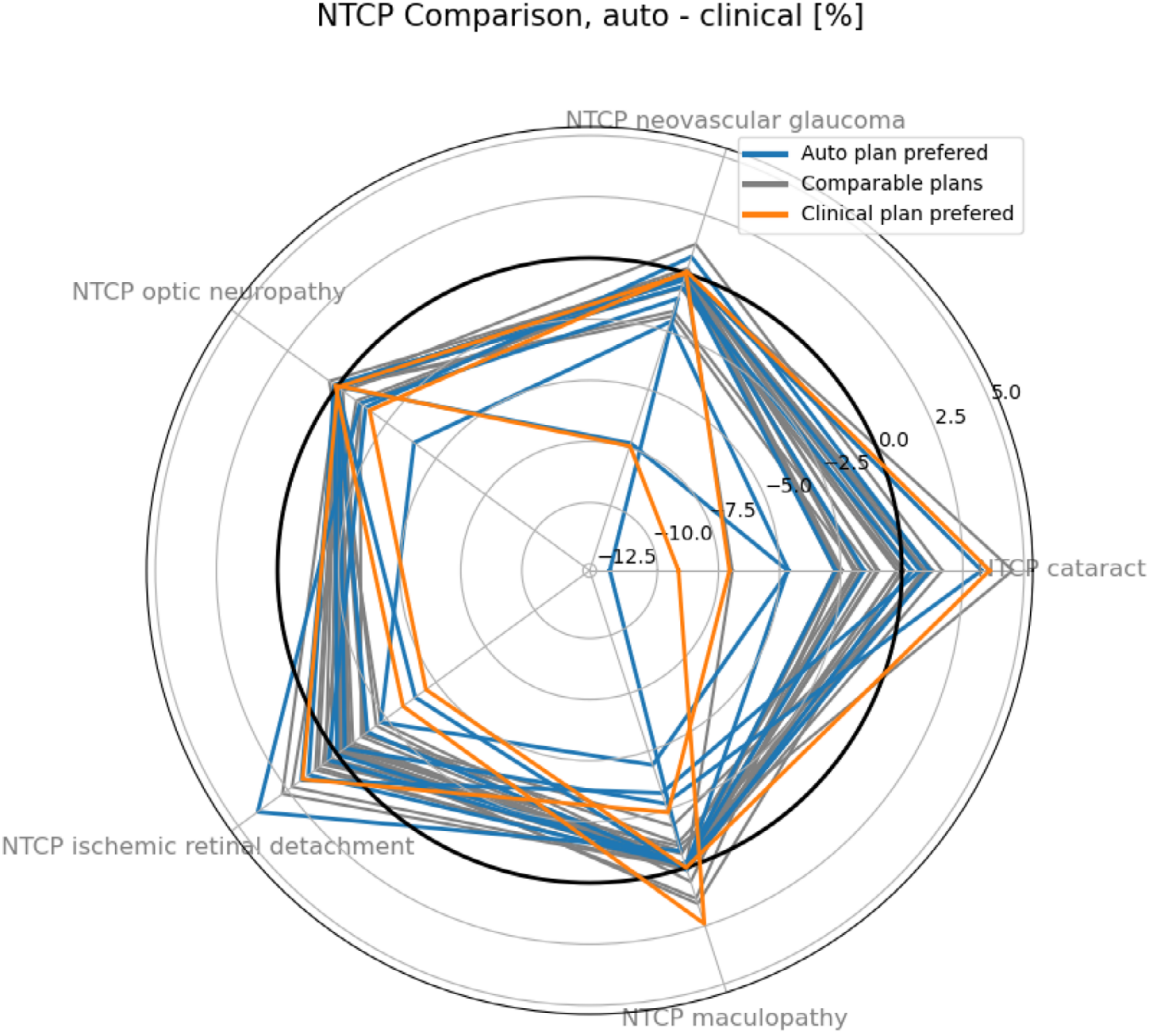
NTCP difference comparison between auto and clinical plans with each curve representing an individual patient. The color represent the preference of the evaluating medical physicists. NTCP, normal tissue complication probability.

**FIGURE 7:**
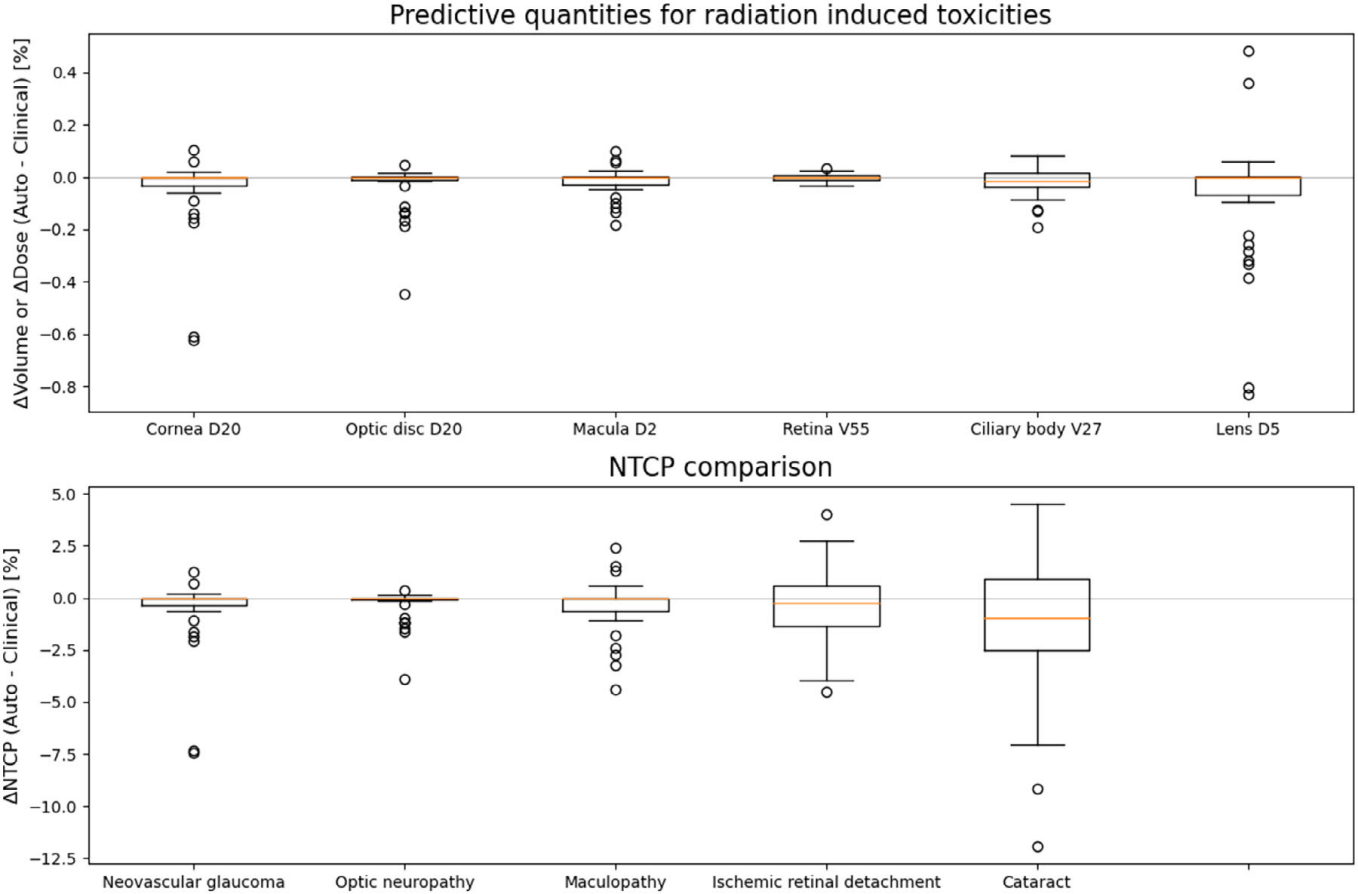
Differences between dose‐volume points related to radiation‐induced toxicity and corresponding NTCP differences between automated and clinical plans. These differences are plotted in isolation without consideration of their inter‐connectivity or trade‐offs. NTCP, normal tissue complication probability.

The preferences of the two medical physicists are shown per patient for the NTCP metrics as a spider plot in Figure 6 and tabulated in cross‐tabulation Table 3 together with the numerical NTCP statistics. In addition, the numerical NTCP analysis is represented in box‐whisker diagrams for both clinically relevant dose‐volume points and NTCP metrics in Figure 7. The medical physicists evaluated the automated plans, rating them as either preferred or comparable in 92% of patient cases, with 25% of these cases being rated as preferred over the clinical plans. In 67% of the cases, the automated plans were judged clinically comparable to the clinical plans by the physicists, based on a qualitative assessment that considered NTCP maps, cost maps, and clinical constraints. The numerical NTCP analysis indicates that the automated plan is either improved or comparable in 69% of patient cases. An improvement, as previously defined, was observed in 47% of patient cases. Among the six numerically categorized trade‐off patient plans, the auto solution is preferred for three patients, while the clinical plan is preferred for three patients. All five plans being designated as inferior were nevertheless still clinically acceptable.

**TABLE 3:** Distribution of preference and numerical based assessments between automated and clinical plans.

		Numerical NTCP statistics				
		Improved	Neutral	Trade‐off	Inferior	Sum
Preference	Auto	5 (13.9%)	1 (2.8%)	3 (8.3%)	0 (0.0%)	9 (25.0%)
	Comparable	12 (33.3%)	7 (19.4%)	0 (0.0%)	5 (13.9%)	24 (66.7%)
	Clinical	0 (0.0%)	0 (0.0%)	3 (8.3%)	0 (0.0%)	3 (8.3%)
	Sum	17 (47.2%)	8 (22.2%)	6 (16.7%)	5 (13.9%)	36 (100%)

The differences between the automatic and clinical treatment plans are illustrated in Figure 7, focusing on individual clinically relevant dose‐volume points and NTCP metrics in isolation without consideration of their inter‐connectivity. The data demonstrate that the automated plan improves over the clinical plan, or remains numerically equivalent, in 67%, 72%, 67%, 61%, and 61% of cases for maculopathy, optic neuropathy, NVG, secondary ischemic retinal detachment and cataract, respectively. The analysis indicates that maculopathy was in the best‐case scenario improved by 4% and a worst‐case increase in probability of 2%. For optic neuropathy, the best improvement was 4%, with no appreciable increase in risk. NVG showed up to 7% improvement and a 1% worst‐case increase. Retinal detachment had up to 4% improvement and a 4% increase in risk. Cataract outcomes saw a 12% improvement and a 5% worst‐case increase.

**FIGURE 8:**
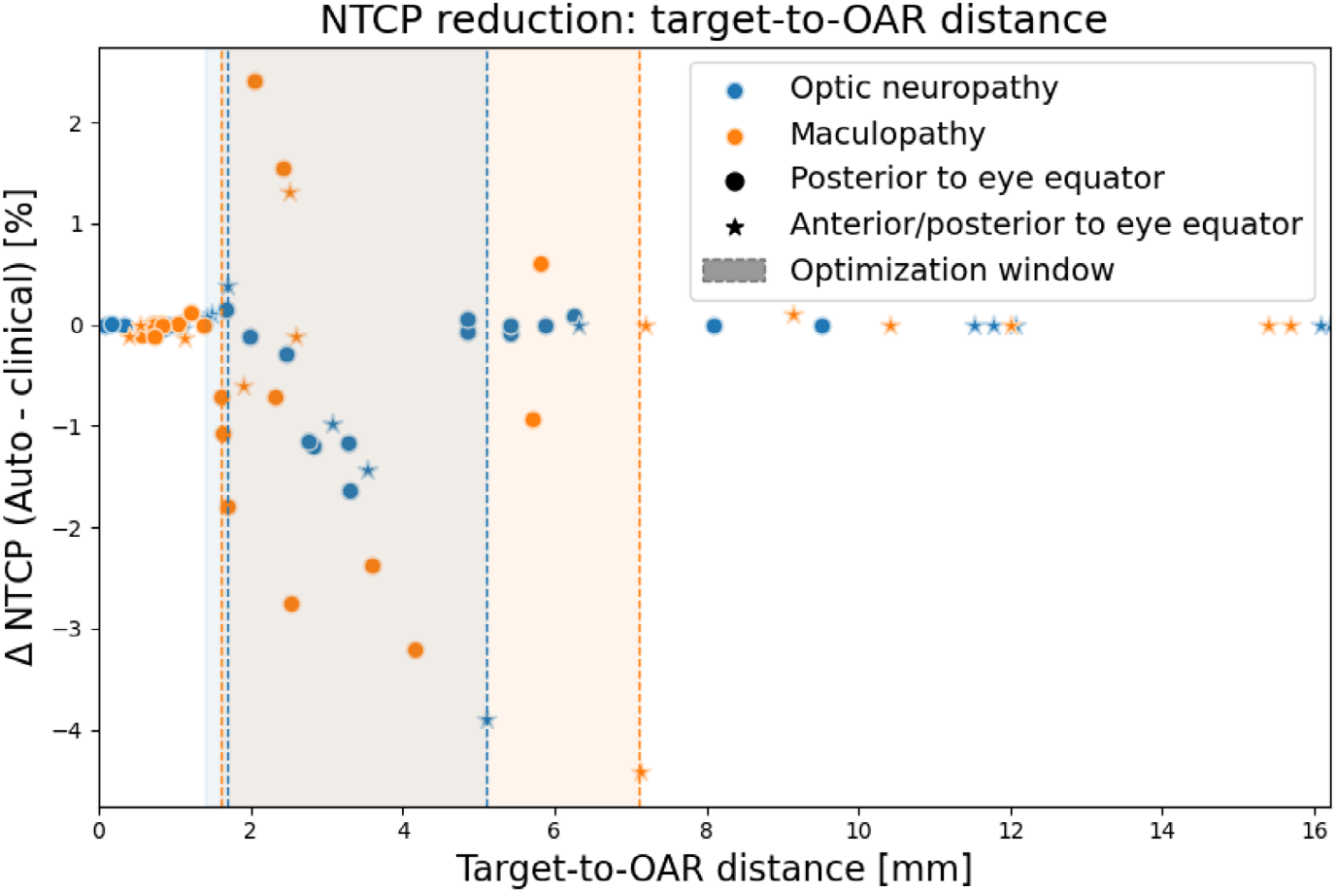
Reduction in NTCP as a function of the distance between the target and the corresponding OAR. The plot highlights an optimization window where the automated treatment plan effectively reduces the risk of toxicity. NTCP, normal tissue complication probability; OAR, organs at risk.

Figure 8 illustrates the reduction in NTCP as a function of the distance between the target and the corresponding OAR. The data reveal an area in which the automated treatment plan could reduce the risk of associated toxicity, starting at approximately 1.65 mm and extending to 5.1 mm for the optic disc and 7.1 mm for the macula. Furthermore, beyond 9.5 mm from the corresponding OAR, only patient cases classified as anterior/posterior to the eye equator were observed. No other consistent relationships between changes in NTCP and the associated OAR or the target's center of mass relative to the eye equator were observed.

## 4 DISCUSSION

This investigation has demonstrated the efficacy of our algorithm in autonomously discerning a suitable eye orientation for treatment using our in‐house developed OCULARIS dose engine. This has been accomplished by correlating the orientation of the treated eye with the onset of five toxicities induced by the treatment beam in healthy tissue and incorporating the predictive factors for these toxicities as penalties in an objective weighted cost function. The parameterization of the tunable cost‐function and NTCP dose‐response curves provides continuous gradients and areas of iso‐values, which can be used by the planner to track iso‐lines of similar healthy tissue sparing in order to identify the gaze fixation point that optimally spares all considered OARs.

Identified fixation points of the automatic algorithm represent a theoretical close to optimal orientation expressed as the orientation associated with the lowest cost. However, the clinical attainability of the orientation identified with the automated optimization algorithm may be hindered by clinical constraints such as avoiding to treat through an eyelid or the patient's ability to gaze according to the treatment plan. Nevertheless, this study indicate that for 92% of the investigated patients, the algorithm achieves comparable or superior preservation of healthy tissue compared to the orientations selected by experienced treatment planners for OPT patients. In nine patients (25%), the automatically created plan was preferred. In three of these 9 cases, the preference for the automated plan was attributed to the fact that the clinical plan represented a compromise between the patient's gaze capability and a fully optimized treatment plan. The patient cases numerically categorized as trade‐off cases indicate scenarios where one favorable NTCP outcome is exchanged for another. In three instances, these cases were preferred by the medical physicist when the trade‐off resulted in improved outcomes for the most critical OARs, namely the optic disc or macula, thereby reducing the risk of inducing optic neuropathy or maculopathy. In five patients (14%), cases numerically classified as inferior were judged clinically comparable by the reviewing physicists. These represented high‐risk cases in which the relative changes in NTCP were small, and the evaluating physicists concluded that such differences did not constitute clinically meaningful inferiority All automated plans were considered clinically acceptable by the investigating medical physicists.

Figure 6 illustrates the relationship between the preferences of the medical physicists and the NTCP differences analyzed for each patient in the study. It highlights the planners' tendency to favor plans that minimize the risk of maculopathy and optic neuropathy, as no automated plans with heightened risks of these toxicities were categorized as preferred. This figure, along with the spans observed in the box‐whisker diagrams in Figure 7, indicates varying potential for optimization between different toxicities. Specifically, the greater variability in the probability of secondary ischemic retinal detachment highlights a more substantial optimization potential compared to NVG, optic neuropathy, and visual acuity related to the macula. Furthermore, an even more pronounced span in sparing potential for the ciliary body V27 and lens D5 underscores the significant potential for optimizing treatment plans to prevent the onset of cataract. The wider span of observed differences in the probability of NTCP for cataract and secondary ischemic retinal detachment can be attributed to several reasons. Firstly, these NTCPs feature steeper response in toxicity risk from increased dose at their respective dose‐volume points. Secondly, the clinical plans are already optimized to spare the macula and optic disc. Thirdly, the risks of maculopathy and optic neuropathy correlate with the proximity to the macula and optic disc to the target.^24^ However, Figure 8 revealed an optimization window, spanning approximately 1.65 to 5.1 mm for the optic disc and 7.1 mm for the macula, where the automated treatment plan could reduce the risk of associated toxicity. In this range, the OAR is sufficiently far away from the target to potentially benefit from an optimized treatment plan, yet not so distant to be devoid of optimization potential. However, attempts to correlate differences between the automated and clinical treatment plans with variations in patient geometry have inherent limitations, as other factors also contribute. The automated plan can only demonstrate superiority if the clinical plan was not fully optimized from the outset, and its effectiveness is further influenced by patient‐specific factors, such as the target extent within the eye and the position of the OAR relative to the distal and lateral falloff regions.

Additionally, the capacity to consistently predict a fixation point has been demonstrated. These findings suggest that, in most cases, the prediction significantly reduces the computational time required by the optimization algorithm to identify a suitable gaze fixation point to just a few minutes, as the optimization time scales with the number of fixation points evaluated. The approach facilitates the evaluation to roughly 35 gaze fixation points, representing a 91.25% reduction compared to the 400 sampling points utilized by Hennings et al.^8^ However, it's important to note that the prediction, while informed by historical patient data at PSI, does not guarantee optimality from an NTCP perspective. The accuracy of the ML predictions could potentially be enhanced through the incorporation of additional labeled NTCP data and a more comprehensive investigation of alternative ML architectures. Its primary use in this context is to guide the initial exploration, with the current implementation serving this purpose effectively. The ML predictions, nevertheless, effectively guide the search for a clinically suitable gaze fixation point. Combined with a termination criterion, this approach can eliminate the need to exhaustively explore all potential fixation points, a methodology previously employed by ref. [8].

The agreement between the ML model's prediction and the minimum‐cost solution in 29 of 36 cases (92%) demonstrates the ML model's alignment with the specific cost function adopted in this study. In the remaining 7 (19%) cases, the minimum‐cost solution was located outside the densely sampled region. The 2D cost maps often exhibited axial or radial symmetry, with symmetric solutions yielding comparable cost values in opposite directions. Of these 7 cases, 6 exhibited symmetric solutions, where the ML prediction and the true cost minimum represented opposite yet nearly equivalent configurations, separated spatially but associated with comparable cost values. There was only one case (3%) in which the ML prediction was clearly suboptimal. This case involved a small tumor, nearly centred on the posterior pole. Here, the ML model proposed a straight‐ahead gaze direction (polar angle close to 0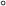), whereas both the clinical choice and the minimum‐cost solution corresponded to a polar angle near 25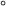. Similarly, in five cases (14%), the clinically applied solution fell outside the ML‐predicted region. Three of these cases corresponded to the symmetric situation previously described. In the remaining 2 cases, the clinical gaze angle was associated with a higher cost value, while the minimum‐cost solution lay within the ML‐predicted region. For both patients, the preference review favored the minimum‐cost solution as the preferred plan. In the NTCP analysis, one patient was categorized as “improved” (reduced toxicity) and the other as a “trade‐off.” These 2 cases illustrate the potential of the proposed tool to quality‐assure the manual solution, potentially leading to improved patient treatment.

The number of fixation points considered to determine the most likely gaze fixation points suitable for treatment located at the periphery, that is, in close proximity to the ML predictions, is approximately 10% of the 441 gaze fixation points investigated in Hennings et al.'s brute force approach, resulting in a proportional reduction in computation time. Figure 3 illustrates the ML algorithm's capacity to learn from the dataset. The features exerting the most significant influence on the prediction of the gaze fixation point is the spatial information concerning the horizontal and vertical positions of the target center of mass in relation to the center of the model in the gaze centered reference frame. The observed learning reflects the ML algorithm's capacity to replicate the planner's intent to choose a gaze that minimizes dose exposure to the anterior segments of the eye by positioning them out of the treatment beam through the selection of a large polar gaze angle. Additionally, the observed learning captures the planner's intention to bring distal targets closer to the surface, thereby reducing the proton range. The ML algorithm also recognizes that certain superficial targets in proximity to the cornea are treated with a gaze directly aligned with the axis of the treatment beam.

Our methodology refines the previous work by ref. [8] by introducing a tunable cost‐function and mapping of NTCP curves to dose calculations. Both contribute to providing actionable gradients of healthy tissue sparing. This approach provides more nuanced association between patient eye orientation and healthy tissue sparing compared to the binary choices for each eye OAR suggested by Hennings et al. Additionally, our approach achieves faster results by avoiding brute‐force sampling despite employing a finer dose calculation voxel grid. It also incorporates a more accurate dose calculation engine based on a physics model,^20^ rather than the simplified fall‐off modeling implemented in EyePlan. Additionally, the proposed guided gaze angle sampling procedure allows for actionable information to be relayed to the planner immediately upon completion of the dose calculation for a given fixation point, rather than requiring exhaustive evaluation of the entire fixation space.

The clinical integration of the methodology proposed in this study appears feasible, with treatment planning guided by calculated 2D cost and NTCP maps. This approach enables the planner to evaluate a wide range of gaze fixation points simultaneously, providing a comprehensive overview of trade‐offs in dose distribution and predicted toxicity. The intended clinical workflow involves the planner initiating the computational process and subsequently reviewing the resulting maps. These outputs are not designed to replace clinical judgment but rather to support it by serving as a decision aid that enhances insight into spatial dose relationships and potential risks. Based on this information, the planner may either accept the automated result or iteratively refine the treatment plan by adjusting the weighting parameters in Equation (2), thereby tailoring the optimization to patient‐specific anatomical and clinical priorities. Alternatively, default weights based on empirical clinical experience can be applied to streamline the process. This integration promotes a more informed and transparent planning strategy, with the potential to reduce subjective biases, improve consistency across cases, and save valuable planning time.

It is important to acknowledge that the NTCP models employed in this work exhibit considerable uncertainties, typically exhibiting standard deviations of a few percent, in some cases, tens of percent. The highest uncertainty involves the probability of retinal detachment, which can reach several tens of percent when the majority of the retinal volumes receive close to the prescribed dose. Moreover, uncertainties inherent to the treatment delivery process further contribute to the overall uncertainty of NTCP predictions. Consequently, small NTCP differences fall within the margin of uncertainty, rendering it challenging to precisely evaluate the likelihood of the treatment beam inducing the investigated secondary toxicity. Moreover, maculopathy was indirectly associated with $D_{2{\%}_{macula}}$ by examining the deterioration in visual acuity. However, it is important to note that visual acuity deterioration is also a symptom of optic neuropathy. As such, the numerical NTCP analysis does not capture the full complexity of the clinical situation, as additional influencing factors such as eyelid position, proton range uncertainty, patients ability to maintain gaze with the gaze fixation point, patient surface, contribute to the clinical decisions. Furthermore, the geometrical models employed in OPT approximate all OARs using simplified geometric surrogates. Given that these OARs are typically small and the dose distributions in OPT exhibit steep gradients, there is inherent uncertainty not only in the actual dose these organs receive but also in the accuracy of the NTCP models derived from the same geometric approximations. The use of a modern TPS incorporating 3D imaging could enhance the precision of these estimates. Moreover, the preference review is limited by the extent of the non‐blind review process. Finally, it is important to recognize that the patient dataset represents only a subset of the treated population and may not fully capture the complexity of patient variations encountered at our facility, as exemplified by numerical findings that may be biased toward cases with targets located posterior to the eye equator.

Future research could concentrate on refining target conformity through the optimization of wedge compensators or by integrating ocular hypertension as a supplementary parameter for minimization. This would involve determining the optimal rotational insert angle, the insert distance across the collimator aperture and the wedge angle, all while ensuring that the distal margin in relation to the target is not compromised. The incorporation of a deformable optic nerve model would represent a valuable advancement in the optimization of OPT treatment planning. Unlike rigid models, which are limited in their ability to capture the true anatomical displacement of this OAR across different gaze orientations, a deformable optic nerve model would more accurately reflect the dynamic positional changes, thereby enhancing the precision of dose attribution.

## 5 CONCLUSIONS

This work signifies a more informed strategy for OPT treatment planning, enhancing both speed and automation while reducing planner biases. The methodology offers treatment planners with direct and intuitive information correlating the orientation of the treated eye with toxicity risks. Our investigation considering the direct application of NTCP models demonstrates that eye orientations for OPT could be automatically generated with a comparable or superior treatment suitability for 92% of the investigated patients.

## CONFLICT OF INTEREST STATEMENT

The authors declare no conflicts of interest.

## Figures and Tables

**FIGURE A1:**
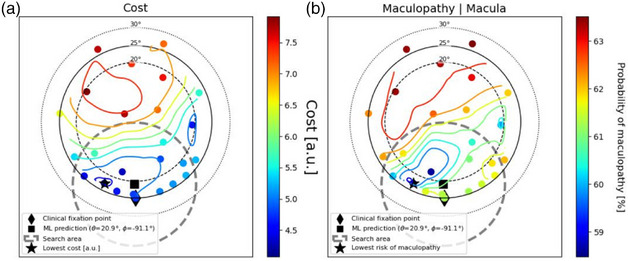
Investigated fixation points for the first patient example are color‐coded based on their respective cost (a) or probability of maculopathy (b).^13^ The target of this patient is located in the right eye superiorly relative to the center of the eye in close proximity to the posterior pole. Larger markers denote key candidate gaze fixation points for treatment.

**FIGURE A2:**
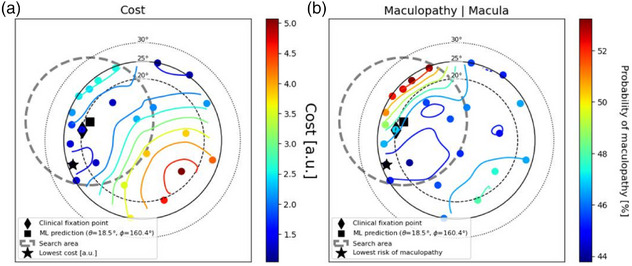
Investigated fixation points for the first patient example are color‐coded based on their respective cost (a) or probability of maculopathy (b).^13^ The target in this patient is located temporally in the left eye, inferior to the center of the eye, and 4.15 mm from the posterior pole. Larger markers denote key candidate gaze fixation points for treatment.

## APPENDIX A

The appendix provides supplementary material in terms of additional patient examples that enhances the understanding of the primary research findings.

### A.1 Additional patient examples

Two additional patient examples are presented in Figure A1 and A2.
